# Topography-driven movement of biomolecular condensates

**DOI:** 10.1371/journal.pone.0345319

**Published:** 2026-04-15

**Authors:** Matthias Pöllmann, Katja Zieske

**Affiliations:** 1 Molecular Biophysics and Living Matter, Max Planck Institute for the Science of Light, Erlangen, Germany; 2 Department of Physics, Friedrich-Alexander Universität Erlangen-Nürnberg, Erlangen, Germany; KIST: Korea Institute of Science and Technology, GERMANY

## Abstract

Biomolecular condensates are assemblies of proteins or nucleic acids that exhibit liquid-like properties and organize intracellular biochemical reactions within many cells. Some condensates require membrane association, and we previously developed an assay to reconstitute biomolecular condensates in the presence of various membrane topographies. However, the effect of membrane topography on the displacement of biomolecular condensates remains incompletely understood. Here, we studied the movement of biomolecular condensates on lipid membrane-clad microstructures in a cell-free assay. We observed movements perpendicular to microstructured surfaces opposing gravitational force for untethered condensates. Increased membrane attachment reduced the number of such movements. These movements were observed for liquid-like condensates as well as for more viscous condensates, on surfaces with grooves and in cylindrical microstructures with varying diameter. A passivating PEG-coating of the microstructures also enabled movements of the condensates perpendicular to microstructured surfaces, further confirming the role topography of modulating passive condensate movement. An increased wetting of condensates on microgrooves led to the formation of elongated condensates which exhibit a coordinated sideward movement upon fusion. Our results indicate that membrane topographies, in combination with membrane attachment patterns, regulate passive biomolecular condensate movement.

## Introduction

Biomolecular condensates are assemblies of proteins or nucleic acids within the intracellular space that regulate cellular function by compartmentalizing biochemical reactions [1]. These condensates form through liquid-liquid phase separation, a process driven by intrinsically disordered domains or weak interactions of multivalent protein domains [2,3]. Although biomolecular condensates are not enclosed by a lipid membrane and are thus also referred to as membrane-less organelles, various biomolecular condensates interact with cellular membranes [4–6]. Upon signaling events such as phosphorylation, some condensates nucleate directly at membranes and remain membrane-associated [2,7,8]. Others exhibit transient membrane binding [9,10]. By interacting with lipid membranes, condensates regulate essential processes, including signal transduction and autophagy [11–13].

The proper localization of biomolecular condensates is critical for cellular function [14]. One mechanism of condensate repositioning involves dissolution and re-condensation at specific cellular locations. This mechanism is important for P granules, which accumulate asymmetrically at the posterior of C. elegans germ cells to regulate asymmetric division [10]. In contrast, RNA granules are actively transported over long distances along microtubules by hitchhiking on lysosomes, ensuring localized protein synthesis far from the nucleus [9]. Lastly, Dishevelled proteins, after forming condensates in the cytosol, are recruited to the plasma membrane upon binding conformationally changed membrane proteins to initiate signaling cascades [5,15]. While several mechanisms governing the movement and localization of biomolecular condensates have been described, the full range of processes underlying the directional movement of biomolecular condensates remains to be elucidated [16].

To investigate the physical principles governing condensate behavior, cell-free reconstitution experiments provide a powerful approach, offering precise control over condensate formation and composition. In addition, these assays provide opportunities to mimic condensate-membrane interactions through defined lipid compositions of model membranes [17–19] and modulation of salinity or membrane charge [20]. Such parameters have been varied to study the wetting phenomena of biomolecular condensates. However, while salt concentrations affect not only the wetting of condensates, but simultaneously other characteristics [21], condensate-interacting lipids enable a more physiological approach for studying condensate wetting on lipid membranes.

Although the biochemical parameters governing the membrane recruitment of biomolecular condensates [2,20,22] and the deformation of membranes by biomolecular condensates [19,23,24] have been extensively studied, the influence of membrane topography itself is less well characterized. Most cell-free assays for studying the wetting of lipid membranes by biomolecular condensates use planar or spherical model membrane systems. However, cellular membranes feature protrusions and microdomains [25]. Therefore, we previously developed an assay to reconstitute biomolecular condensates on topographically structured membranes and demonstrated that membrane topography affects the shape and localization of biomolecular condensates through capillary forces mediated by wetting and interfacial tension [18].

Biomolecular condensates exhibit liquid-like properties, including fusion, fission, and molecular exchange with their surroundings and within the condensate [10,26]. These properties suggest that principles from droplet physics may also apply to biological condensates. For example, the behavior of biomolecular condensates on structured lipid membranes may be similar to the behavior of water droplets on microstructured surfaces. In the presence of microstructured surfaces the motion of water droplets can be guided [27], with wetting properties influencing the droplet movement [28]. The movement of water droplets, widely studied in material sciences and research on anti-fouling systems, demonstrates how surface topography can influence liquid dynamics [29,30]. We hypothesized that, similar to water droplets responding to surface topography, the topography of lipid membranes may direct the movement of biomolecular condensates.

Here, we used a cell-free assay to study the movement of biomolecular condensates on topographically structured lipid membranes. We show that condensates within membrane microgrooves move perpendicular to the microstructured surface, overcoming gravitational force, once they have grown beyond a critical size. In the following, we refer to this type of movement orthogonal to the microgrooved surface as ‘lifting’. We demonstrate that this behavior primarily occurs in the absence of membrane interaction and that the ‘lifting’ phenotype was still retained, when condensates were induced to display a more gel-like behavior. Increasing the concentration of condensate-interacting lipids led to membrane wetting, causing condensates to remain preferentially within the microgrooves. Furthermore, the diameter of microcylindrical indentations determines the condensate size at which these movements occur. Notably, condensates on PEG-coated microstructures exhibit similar movement as untethered condensates on lipid membranes. Stronger wetting of condensates on membrane-clad microgrooves resulted in the formation of elongated condensates, which exhibited a zipper-like sideward movement towards the grooves. This movement was induced by condensate fusion. Our observations indicate that geometries of lipid membrane topography affect the movement of liquid biomolecular condensates. Wetting via protein-membrane interactions may thus act as a switch that directs biomolecular condensates into or out of cellular membrane protrusions.

## Results and discussion

**Fig 1:**
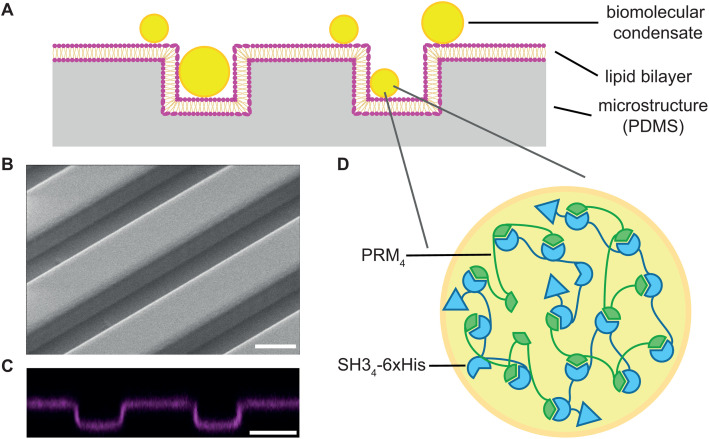
Cell-free assay for the characterization of biomolecular condensates on topographically structured membranes. (A) Schematic representation of the experimental system: Microstructured PDMS surfaces were clad with a lipid membrane. Liquid biomolecular condensates were assembled in the buffer solution above these lipid membranes. (B) Scanning electron microscopy image of microstructured PDMS surface. (C) Fluorescent laser scanning confocal microscopy image of a supported lipid bilayer composed of DOPC and 0.05 mol% Fast DiI. (D) Schematic illustration of biomolecular condensate composition. Biomolecular condensates were composed of the two proteins PRM_4_ and SH3_4_-6xHis. The SH3 domains interact weakly with PRM domains, and based on this interaction, the liquid biomolecular condensates were assembled. Scale bars: 10 µm.

Membrane topography and mechanical confinement play essential roles in the coordination of cellular processes and the organization of biomolecular condensates. For instance, during cellular migration through small pores, diameter constrictions of a membrane-enclosed nuclear volume and chromatin deformations influences the shape and formation of biomolecular condensates [31]. To study the effects of membrane topography on the dynamic localization of biomolecular condensates in a well-defined system and in the absence of additional cellular complexities, we employed a cell-free assay. Specifically, we reconstituted proteins that assemble into biomolecular condensates on microstructured PDMS surfaces coated with lipid membranes (Fig 1A). Thereby, we selected an array of grooves as the topographical feature, because the bottom of these grooves represents an environment where protein condensates are confined by two walls and it covers a large proportion of the total area. The grooves were 7 µm deep and 10 µm wide. Condensates within the grooves were able to grow through lateral fusion and enable facile analysis of individual biomolecular condensates. The PDMS microgrooves were fabricated using photolithography and soft molding. To visualize the surface topography and confirm the structural integrity of the microgrooves, we performed scanning electron microscopy (Fig 1B). Lipid bilayers were generated via vesicle fusion and composed of DOPC, supplemented with 0.05 mol% Fast-DiI. Where specified, the membranes were supplemented with varying concentrations of the functional lipid DGS-NTA, which interacts with histidine tags of purified proteins. Laser scanning confocal imaging was used to confirm the presence of continuous lipid membranes that adopted the topography of the PDMS microgrooves (Fig 1C, S1). Lipid membranes were stable for at least 26 h with minor reduction of fluidity after 72 h (S2 Fig). To induce the formation of biomolecular condensates, we added the two multivalent proteins PRM_4_ (40 µM, 5% labelled with AF488) and SH3_4_-6xHis (40 µM). PRM_4_ consists of four identical proline-rich motifs (PRMs) of the protein ABL1. The protein SH3_4_-6xHis contains four identical SH3 domains derived from NCK1 (Fig 1D) [2] and a c-terminal histidine-tag. The histidine-tag facilitates membrane binding via interactions with the functional lipid DGS-NTA and bridges proteins in the presence of dissolved Ni^2+^.

**Fig 2:**
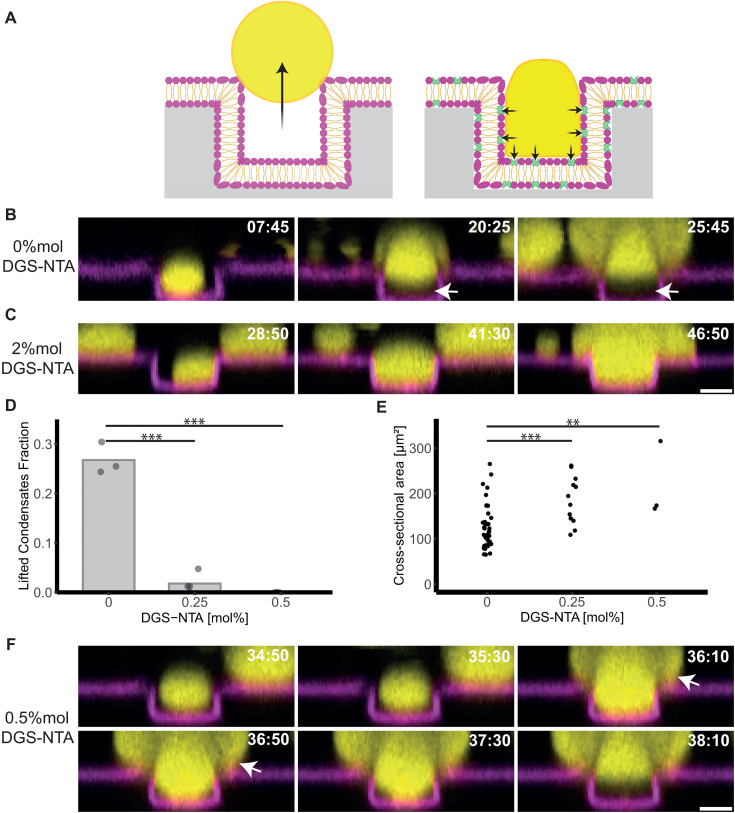
Lifting of biomolecular condensates in microgrooves against gravity is determined by the absence of membrane attachment. (A) Schematic representation of biomolecular condensate dynamics within microstructured grooves in the absence (left: lifting) and presence (right: remaining at the bottom of the microgrooves) of DGS-NTA – a membrane component that binds to the histidine tag of SH3_4_-6xHis. (B, C) Confocal time-lapse images of biomolecular condensates (yellow) within membrane grooves (magenta) in the absence (B) and presence (C) of 2 mol% DGS-NTA. The two condensates were selected for their similar size and similar time intervals, allowing for a direct comparison of their behavior in the presence of 2 mol% DGS-NTA. Independent samples for each condition: n = 3. Scale bar: 5 µm. (D) The fraction of condensates (larger than 1 µm^3^) which lifted within the grooves in the absence of DGS-NTA and in the presence of 0.25 mol% and 0.5 mol% DGS-NTA. Bar plot: Average number of biomolecular condensates displaying lifting. Independent samples: n ≥ 3. Two-tailed t-test: p < 0.001 (0% vs. 0.25% DGS-NTA) and p < 0.001 (0% vs. 0.5% DGS-NTA). (E) The cross-sectional areas of condensates after detachment from the groove bottom was determined. Cross-sectional area measurements were performed 3 µm above the top of the PDMS microstructures. Two-tailed t-test: p < 0.001 (0% vs. 0.25% DGS-NTA) and p = 0.0051 (0% vs. 0.5% DGS-NTA). Number of measured condensates across three independent samples per condition: 47 (0% DGS-NTA), 12 (0.25% DGS-NTA), and 3 (0.5% DGS-NTA). (F) Confocal time-lapse images showing condensate growing in a mushroom shaped volume (white arrow) and subsequent detachment in the presence of 0.5 mol% DGS-NTA. Asterisks indicate statistical significance: p < 0.05 (*), p < 0.01 (**), and p < 0.001 (***). Protein concentrations in all experiments: 40 µM PRM_4_, 40 µM SH3_4_-6xHis. Lipid bilayer composition: DOPC, 0.05 mol% Fast-DiI and 0–2 mol% DGS-NTA (as indicated). Time points refer to the time point (t = 0) when phase separation was induced by supplementing the sample with proteins.

To explore how confinement from two sides affects the dynamics of biomolecular condensates, we performed time-lapse confocal microscopy on biomolecular condensates within membrane-clad microgrooves. Thereby, we observed lifting of large biomolecular condensates within the grooves in the absence of membrane attachment (Fig 2A, 2B, video S1). Initially, these condensates grew in the buffer solution through fusion and incorporation of protein monomers, and sedimented toward the microstructured bottom of the sample chamber, where the biomolecular condensates continued to grow. Condensates maintained a spherical morphology, and when their diameters exceeded the groove width, the condensates moved perpendicular to the sample surface against gravity. The condensates regained their spherical shape above the confining microgrooves.

The movement of biomolecular condensates can be attributed to an interplay of various forces. In solution without any confinement, gravitational force drives the sedimentation due to the higher density of biomolecular condensates compared to the buffer solution [32]. Thereby, the interfacial tension stabilizes their spherical shape during growth. As the volume of the condensate increases, the condensate fraction confined within the microstructure forms a strongly curved morphology compared to the less confined upper fraction. This difference in curvature generates a capillary pressure difference across the condensate. When the force generated by this capillary pressure difference exceeds the opposing gravitational and adhesive forces, the condensate moves out of confinement into the less restricted space. The observed lifting of biomolecular condensates closely resembles the motion of water droplets within superhydrophobic grooves, although such water droplets moved at air interfaces and microgrooves were an order of magnitude larger [33]. In addition, swelling and shrinking of biomolecular condensates with the same protein content [32] suggests increased compressibility of condensates compared to pure water droplets which may influence internal pressure differences.

Next, we assessed how increased membrane wetting affects condensate dynamics. Our previous work demonstrated that DGS-NTA, which binds strongly to histidine-tagged condensate components, promotes membrane wetting by the condensates and increases the membrane contact angle as compared to condensates on membranes without DGS-NTA (S3 Fig) [18]. When we supplemented lipid membranes with 2 mol% DGS-NTA, lifting of condensate was no longer observed (Fig 2A, 2C, video S1). When we decreased the DGS-NTA concentration to 0.5 mol%, we still did not observe any lifting within 35 minutes, indicating strong adhesion of the condensates to the membrane. When we decreased the DGS-NTA concentration further to 0.25 mol%, some lifting condensates were observed (Fig 2D). However, without DGS-NTA the frequency at which lifting occurred, was significantly higher (Fig 2D).

After longer incubation times of 45 min, larger condensates formed, and lifting condensates were observed even in the presence of 0.5 mol% DGS-NTA. We measured the cross-sectional area of condensates after detachment from the bottom of the grooves (Fig 2E), confirming that lifting condensates were on average smaller in the absence of DGS-NTA, as compared to lifting condensates in the presence of DGS-NTA. In the presence of 0.5 mol% DGS-NTA, the condensates grew into a mushroom-shaped volume when they exceeded the confined space. They maintained this shape for tens of seconds, while remaining attached at the bottom until they finally detached from the bottom surface of the microgrooves (Fig 2F). Our data indicate that the size of condensates required for liftings increases with increasing membrane attachment. At the contact site between the lipid membrane and the biomolecular condensate, the histidine-nickel interactions induce wetting, because increasing the DGS-NTA concentration within the lipid membrane enhances the density of potential binding sites. This increased membrane binding results in the requirements for a larger capillary pressure difference for lifting condensates against gravity, which is achieved by an increased volume of the condensate. This observation is in agreement with simulations of water droplets on microgrooves where wetting properties were modulated [34]. Cells can modulate binding between biomolecular condensates and cellular membranes [35], potentially using wetting regulation as a switch to control condensate movement. Notably, while some biomolecular condensates reach micrometer scales similar to the condensates in our assay [10], many condensates in cells are even smaller. For small condensates, the viscosity of the surrounding fluids may be an additional parameter that influence their dynamics.

**Fig 3:**
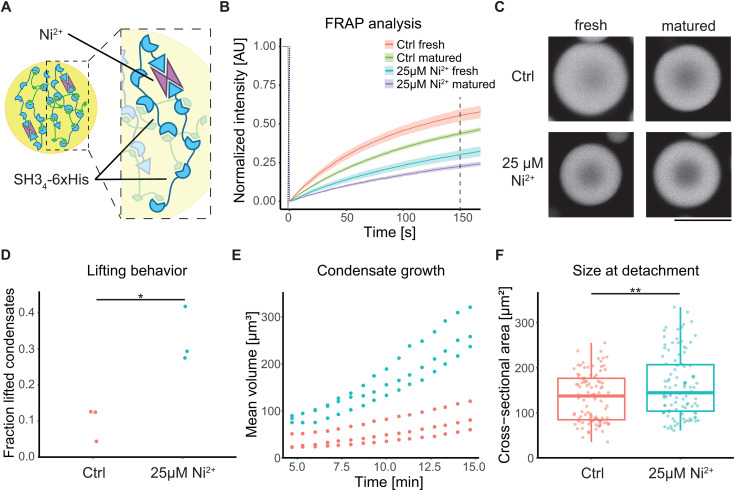
Ni^2+^-induced viscosity increase preserves lifting. (A) Schematic representation of Ni^2+^ chelating His-tags from two independent SH34-6xHis proteins. (B, C) FRAP assay of biomolecular condensates in the absence and presence of 25 µM Ni^2+^ and with incubation for 1–1.5 h (fresh) or 20–24 h (matured) after bleaching a 5 µm diameter circle in a 10–13 µm sized biomolecular condensate. Line plot shows normalized mean intensity ± standard deviation over time. Dotted line: photobleaching; dashed line: time point when exemplary images (C) were taken. n ≥ 8 from three independent samples per condition (D) The fraction of lifted condensates (larger than 1 µm^3^) 20 min after induction of phase separation by combining both proteins in the sample in the presence and absence of 25 µM Ni^2+^. Two-tailed t-test: p = 0.018, n ≥ 342 analyzed condensates per replicate (E) Kinetics of condensate growth as mean volume over time. (F) Maximal cross-sectional area of biomolecular condensates when they detach from microstructure’s bottom in the absence and presence of 25 µM Ni^2+^. Two-tailed t-test: p < 0.001. Independent samples: n = 3 for (D-F). Asterisks indicate statistical significance: p < 0.05 (*), p < 0.01 (**), and p < 0.001 (***). Protein concentrations in all experiments: 40 µM PRM_4_, 40 µM SH3_4_-6xHis. Lipid bilayer composition: DOPC, 0.05 mol% Fast-DiI. Scale bar: 20 µm (C).

These results demonstrate that the movement of biomolecular condensates out of confinement is driven by their interfacial tension and the lack or low abundance of membrane linkers. To determine how biomechanical properties may affect the lifting phenotype, we induced increased protein interactions to mimic a more gel-like phenotype. Shear relaxation, which governs shape recovery, occurs faster in more liquid-like biomolecular condensates [36]. We therefore hypothesized that more gel-like condensates are more plastically deformed by confining microstructures before lifting. This more connected condensate state was induced by adding 25 µM Ni^2+^ to the buffer. Ni^2+^ can bridge two His-tags and thereby act as additional crosslinker between two SH3_4_ proteins within the condensates (Fig 3A) [37,38]. Unlike other condensates such as FUS [39], FRAP experiments indicate that PRM_4_/SH3_4_ condensates exhibit a rather weak liquid-to-gel transition upon aging. Their viscosity is however increased in the presence of 25 µM Ni^2+^, as indicated by a reduced internal mobility observed in FRAP experiments (Fig 3B, 3C) and they display slower fusion dynamics (S4 Fig) [36]. In the presence of Ni^2+^ and thus, under more crosslinked condensate conditions, we still observed movement against gravity out of confinement (Fig 3D). In addition to viscosity, condensate growth also increased in the presence of Ni^2+^, resulting in a larger number of condensates lifted 20 min after induction of phase separation (Fig 3D, 3E and S5 Fig). To minimize the confounding effect of growth in our analysis on lifting, condensate sizes at the time point of detachment were compared, revealing that lifted condensates are slightly larger in the presence of Ni^2+^ (Fig 3F). This supports the hypothesis that more gel-like condensates are more plastically deformed during transient confinement, requiring them to grow larger before they are lifted out of the confined space. Overall, these findings indicate that the movement out of confinement is a property of liquid-like condensate with varying biomechanical properties.

**Fig 4:**
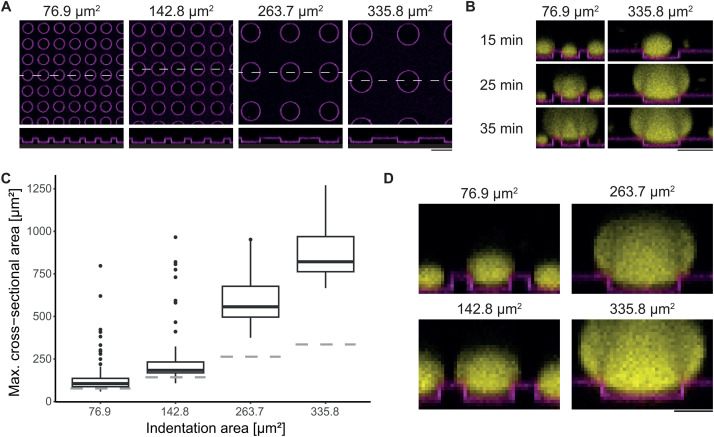
Microstructure size determines condensate detachment size. (A) Confocal xy- and xz-images of lipid bilayers on microstructured cylindrical indentations with areas of 76.9 µm^2^, 142.8 µm^2^, 263.7 µm^2^, and 335.8 µm^2^ at a constant depth of 7 µm. (B) Representative confocal time-lapse images of biomolecular condensates on cylindrical indentations with an area of 76.9 µm^2^ and 142.8 µm^2^. The time stamp refers to the time point when proteins were added to the sample volume. (C) Maximal cross-sectional area at the timepoint of detachment from the bottom of the cylindrical indentation. Median cross-sectional area at detachment per structure: 106.0 µm^2^ (interquartile range (IQR): 87.7–136.6 µm^2^; 76.9 µm^2^ indentation), 183.8 µm^2^ (IQR: 168.7–232.7 µm^2^; 142.8 µm^2^ indentation), 556.7 µm^2^ (IQR: 496.2–677.4 µm^2^; 263.7 µm^2^ indentation) and 821.3 µm^2^ (IQR: 762.8–969.2 µm^2^; 335.8 µm^2^ indentation). Number of measured condensates across three independent samples per structure: 144 (76.9 µm^2^ indentation), 94 (142.8 µm^2^ indentation), 52 (263.7 µm^2^ indentation) and 31 (335.8 µm^2^ indentation). Grey dashed line indicate the indentation area. (D) Confocal images showing lifted condensates for varying indentation areas. Protein concentrations: 40 µM PRM_4_, 40 µM SH3_4_-6xHis. Lipid bilayer composition: DOPC, 0.05 mol% Fast-DiI. Scale bar: 20 µm (A, B), 10 µm (D).

In cells, the geometry and size of confining structures such as membrane protrusions displays a large range of phenotypical variations. To address how geometrical parameters affect lifting of condensates, we fabricated membrane-clad microstructures with cylindrical indentations of varying areas (76.9 µm^2^, 142.8 µm^2^, 263.7 µm^2^, and 335.8 µm^2^) (Fig 4A). Unlike microgrooves, which restrict movement laterally to one axis, cylindrical indentations confine condensates laterally in all directions. Consistent with condensate behavior on microgrooves, condensates sedimented and grew at the bottom of the cylindrical indentations (Fig 4B). In cylindrical indentations with a smaller area, condensates detached from the bottom and lifted at a smaller size as compared to indentations with a larger area (Fig 4C, 4D). Specifically, detachment occurred when the cross-sectional area of the condensate had grown slightly above the area of indentation for 76.9 µm^2^ and 142.8 µm^2^ indentations. In contrast, for larger indentations, where the radius of the structure is larger than its depth (9.2 and 10.3 µm compared to 7 µm for 263.7 µm^2^ and 335.8 µm^2^ large indentations), the condensates detached only when they achieved significantly larger cross-sectional areas than the indentations themselves. This suggests that as the confining boundaries become wider, condensates must grow larger and achieve a lower contact angle relative to the planar top surface of the microstructure to completely detach from the bottom. These observations highlight the critical role of geometrical constraints in topography-induced movement of biomolecular condensates.

**Fig 5:**
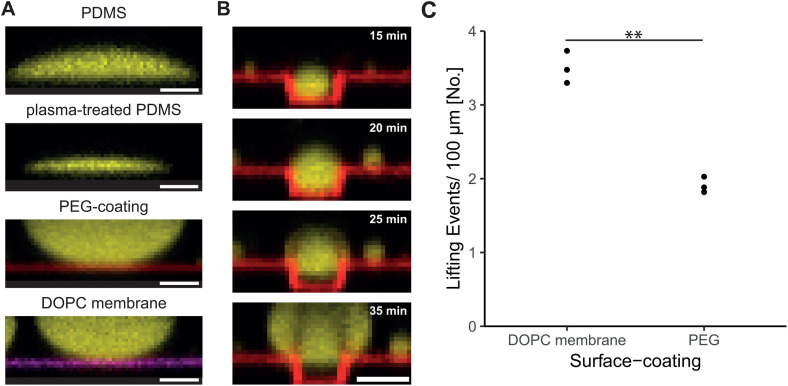
Lifting of biomolecular condensates on PLL-g-PEG coated microgrooves. (A) Confocal images of biomolecular condensates on PDMS, plasma-treated PDMS, PLL-g-PEG/Atto633-coating (red) and DOPC lipid membrane with 0.05 mol% Fast-DiI (magenta). Exemplary images of condensates. n ≥ 3 independent samples for each condition. Scale bar: 20 µm. (B) Confocal time-lapse images of biomolecular condensate (yellow) within PEG-coated microgroove (red). The time point of adding proteins to our samples was defined as t = 0 min. Scale bar: 20 µm. (C) The number of lifting events was quantified per 100 µm groove length. Two-tailed t-test: p = 0.0017. Asterisks indicate statistical significance: p < 0.05 (*), p < 0.01 (**), and p < 0.001 (***). Protein concentrations in all experiments: 40 µM PRM_4_, 40 µM SH3_4_-6xHis.

We observed that low adhesion of condensates to lipid membranes leads to more lifting events within membrane-clad microgrooves as compared to high adhesion to membranes. We therefore hypothesized that condensates on passivated surfaces may also display directed movements out of confined space. As PRM_4_/ SH3_4_ biomolecular condensates show strong wetting on untreated and plasma-treated PDMS (Fig 5A), we additionally coated the plasma-activated PDMS surface with PLL-g-PEG/Atto633. PLL-g-PEG forms a thin homogeneous layer with low adhesion to proteins and biomolecular condensates [40,41] (Fig 5B). Similar to membrane-clad microstructures, we observed a lifting of condensates in PEG-coated grooves when the condensate size exceeded the groove boundaries (Fig 5B). The number of lifting events is reduced compared to samples with a DOPC lipid membrane (Fig 5C), suggesting a stronger interaction of the biomolecular condensates to the PEG-coated surface compared to the DOPC lipid membrane. These results show that a low-adhesive surface coating is sufficient to direct the movement of condensates in confinement. Thus, low-adhesive surface coatings, such as lipid membranes as well as PEG-coated surfaces [42] can be applied to study the dynamics of biomolecular condensates in confined space.

**Fig 6:**
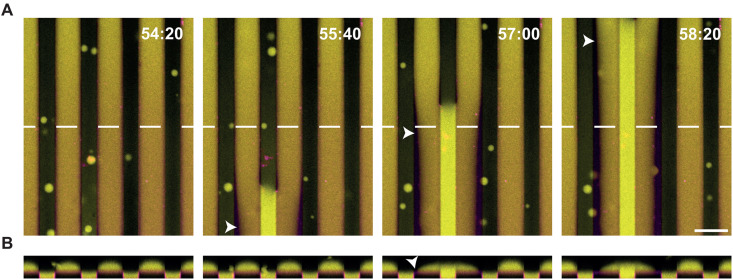
Lateral zipper-like movements of elongated biomolecular condensates on microgrooves are observed in the presence of 4% DGS-NTA. (A) Time-lapse confocal microscopy images of biomolecular condensates (yellow) in the presence of 4 mol% DGS-NTA. Elongated biomolecular condensates assembled within the valleys and at the upper level of membrane-clad PDMS surfaces with microgrooves (magenta). When condensates on the upper PDMS-level fused with condensates in the microgroove valleys, the condensates on the upper PDMS-level exhibited a lateral shift toward the microgroove. This resulted in a condensate-free area along the opposite edge of the upper PDMS level (white arrowheads). (B) Side view of biomolecular condensates on the same membrane surfaces along the white dotted line in (A). Protein concentrations: 40 µM PRM_4_ and 40 µM SH3_4_-6xHis. Lipid bilayer composition: DOPC, 0.05 mol% Fast-DiI, 4 mol% DGS-NTA. Independent samples: n = 3. Scale bar: 20 µm.

Next, we characterized how even stronger wetting influences the behavior of biomolecular condensates. For this, we supplemented the lipid membranes with 4 mol% DGS-NTA. Under these conditions, condensates in the grooves and those on the flat surfaces between them fused into bigger condensates, respectively. These fusions resulted in large, elongated condensates in the grooves and on the upper, flat surfaces between them (Fig 6A, 6B). Initially, these condensates remained separated (Fig 6B, left). However, when a condensate within a groove grew and reached the microstructure rim, it fused with adjacent condensates on the upper surface. In some instances, we observed fusion only on one side. Fusion was initiated at one contact point and then propagated along the length of the elongated biomolecular condensate (Fig 6, S2 video). The fusion process progressively pulled the corresponding condensates on the upper surface towards the condensate within groove, resembling a zipper-like motion along the microgrooves.

The formation of elongated biomolecular condensates results from the interplay of microstructure, interfacial tension, and the strength of membrane attachment. We showed previously that other membrane-clad microstructures generate ordered patterns of biomolecular condensates [18]. The present assay allowed us to generate elongated condensates and to study their planar zipper-like displacement. The observed coordinated movement may in the future provide insights into the mechanisms of physiologically relevant membrane-bound condensates, such as ZO-1 condensates in tight junctions [43].

In summary, we have shown that the motion of biomolecular condensates can be induced by interfacial tension and confinement. The behavior of biomolecular condensates in membrane-clad microgrooves thereby resembles the movement of water droplets on superhydrophobic microstructures and can be modulated by wetting of the condensates. Given the diverse topographical features of cellular membranes, these results may provide insights into the dynamic localization of biomolecular condensates within membrane-confined regions of the cell.

## Materials and methods

### Preparation of vesicles and supported lipid membranes

Supported lipid membranes were generated by vesicle fusion. The lipids DOPC and 18: 1 DGS-NTA (Ni) (dissolved in chloroform; Avanti Polar Lipids) and 0.05 mol% Fast DiI (dissolved in ethanol, ThermoFisher) were mixed in a glass vial. The lipid solution was dried under a nitrogen stream and then desiccated under vacuum to remove residual solvents. The lipid film was resuspended in membrane buffer (pH = 7.5, 25 mM Tris, 150 mM KCl) to a concentration of 4 mg/ml, incubated at 37°C for 30 min and sonicated. The vesicle solution was aliquoted, frozen and thawed only once.

To generate supported lipid membranes, the vesicle solution was diluted to 0.5 mg/ml in membrane buffer, supplemented with 4 mM CaCl₂ and added to a custom-made reaction chamber with a bottom consisting of a PDMS layer with microstructures. The lipid solution was then incubated at 37°C for 30 min and a lipid bilayer membrane assembled. The residual vesicles were removed by repeated dilution of the solution above the membrane. Finally, membrane buffer was replaced with KMEI buffer.

### Preparation of PDMS structure

PDMS microstructures were prepared as described previously [18]. The two-dimensional geometry of the microstructures was defined by patterns on a chrome mask. Approximately 7 µm high photoresist structures (mr-DWL 5, micro resist technology) were fabricated on silicon wafers by photolithography. The wafers were then treated with trimethylchlorosilane (Sigma-Aldrich). Microstructured PDMS surfaces were fabricated by soft molding. PDMS (Sylgard184, monomer to crosslinker ratio 10:1, Dow Corning) was degassed and poured on the silicon wafers. Glass coverslips were pressed through the liquid PDMS onto the microstructures. The PDMS was cured by incubation at 60°C for at least 3 hours. The glass coverslips with a layer of microstructured PDMS were carefully recovered. Surface structures were visualized using scanning electron microscopy (GeminiSEM 560, Zeiss). Before assembling lipid membranes or PEG coating, PDMS surfaces were treated with oxygen plasma at 0.3 mbar for 1 min to increase wettability and a plastic cylinder was glued on top of the PDMS surface. For PEG coating of the microstructure, 0.1 mg/ml PLL-g-PEG/Atto633 (PLL(20)-g[3.5]-PEG(2)/Atto633, SuSoS Surface Technology) in PBS was added, incubated for 1 h at room-temperature and residual PEG was removed by repeated dilution with KMEI buffer.

### Microscopy

Microscopy images were acquired using a laser scanning fluorescence microscope (LSM980, Zeiss) with a 20x objective (Plan-Apochromat 20x/ 0.8, ∞ /0.17, Zeiss). Microscopy experiments were performed at room temperature. Protein assays were performed in KMEI buffer. Protein condensation was induced by mixing 40 µM SH3_4_-6xHis and 40 µM PRM_4_. 5% of PRM_4_ was labelled with AF488. Time measurements started when both proteins had been added to the sample chamber, unless otherwise noted.

### FRAP analysis of condensate fluidity

FRAP experiments were performed on biomolecular condensates on DOPC lipid bilayers with 0.05 mol% Fast-DiI. Circular regions with a diameter of 5 µm were photobleached within condensates (condensate diameter: 10–13 µm) using a 488 nm laser. Fluorescence recovery was monitored by acquiring images in 1.26 s intervals. The fluorescence intensity of the analyzed region was normalized.

### Software

ImageJ/Fiji (v2.14.0/ 1.54f) was used for image analysis [44]. Volume measurements were conducted using 3D Objects Counter [45]. Cross-sectional areas of condensates were measured from central z-stack slices, after ensuring that at least half of each condensate was captured in the z-direction. The plug-in 3D Viewer was used to reconstruct 3D images displayed as volume from z-stack images [46].

Subsequent data analysis and statistical tests were performed in R (v4.3.2) using RStudio (v2023.12.1). Adobe Illustrator (v28.7.1) was used to arrange figures and generate schematics.

## Supporting information

S1 Fig Supported lipid membrane adopting the topography of PDMS microstructures. (A) Fluorescent laser scanning confocal microscopy images of the lipid bilayer, labelled with 0.05 mol% Fast DiI. Images show planar views (top, mid and bottom of the microstructure) and a side view. Scale bars: 20 µm. (B) Side view of a membrane-clad microstructure, with annotations for the levels defined as “top”, “mid” and “bottom”. Scale bar: 5 µm. (C) 3D reconstruction of the lipid bilayer topography, derived from z-stack images obtained via fluorescent laser scanning confocal microscopy.
(PDF)

S2 Fig Supported lipid membrane remains stable for up to 3 days on PDMS surface. (A) FRAP experiments were performed at various time points (30 min, 26 h, 72 h, and 7 d after membrane formation) on membranes composed of DOPC and 0.05 mol% Fast DiI. (B) Fitted FRAP curves and quantification of half-time recovery reveal stable membranes over 26h (t_1/2–30min_: 21.6 ± 1.6 s; t_1/2-26h_: 22.7 ± 1.0 s), with slightly reduced fluidity after 3 days (t_1/2-72h_: 25.8 ± 0.6 s) and more pronounced reduction of fluidity after seven days (t_1/2-7d_: 31.2 ± 2.1 s). Dashed line: time point of bleaching of the membrane. Half-time recovery and line plot show the mean intensity ± standard deviation. n = 9 from three independent samples. (C) Membrane homogeneity is maintained within 72 h, but impaired after 7 d, with white spots and darker areas indicating compromised membrane integrity.
(PDF)

S3 Fig Condensate show increased wetting for higher concentrations of DGS-NTA. (A) Confocal fluorescence microscopy images of biomolecular condensates (yellow), chosen to exhibit an average xy-diameter of 7.7 ± 0.5 µm, formed on DOPC lipid membranes containing 0.05 mol% Fast-DiI (magenta) and supplemented with 0 mol%, 0.25 mol%, 0.5 mol%, or 2 mol% DGS-NTA. Condensates wet the lipid membrane with higher DGS-NTA concentrations with an increasing contact angle (white arrow). Independent samples: n = 3. Protein concentrations: 40 µM PRM_4_, 40 µM SH3_4_-6xHis. Scale bar: 10 µm. (B) Quantification of contact angle using ImageJ’s contact angle plugin. Individual measurements per condensate were depicted (black data points) alongside the mean value ± standard deviation (red).
(PDF)

S4 Fig Biomolecular condensates display slower fusion and relaxation in the presence of 25 µM Ni^2+^. Fusion assay with and without 25 µM Ni^2+^ was performed 70 ± 5 min after condensate formation was initiated by supplementing samples with 40 µM PRM_4_ and 40 µM SH3_4_-6xHis. Image sequence shows representative fusion events for n = 3 independent replicates. The time point t = 0 refers to the onset of fusion of two similar sized condensates. Asterisks indicate the onset, and arrows the end of fusion events. Lipid bilayer composition: DOPC, 0.05 mol% Fast-DiI. Scale-bar: 10 µm.
(PDF)

S5 FigGrowth of biomolecular condensates in the presence and absence of 25 µM Ni^2+^.Boxplots show size analysis of biomolecular condensates larger than 10 µm^3^ in the first 5 to 15 min after their formation in the presence and absence of 25 µM Ni^2+^. The right panel shows boxplots after exclusion of outliers (defined as outside $median\pm \text{1}.\text{5}\text{8}*\frac{interquartile\;range\;(IQR)}{\sqrt{n}}$).(PDF)

S1 Video Biomolecular condensate movement in the absence and presence of 2% DGS-NTA. Time-lapse images were acquired using a confocal laser scanning microscope. In the absence of DGS-NTA, condensates exhibit an upward movement upon exceeding the groove boundaries. In the presence of 2% DGS-NTA, condensates remain confined within the microgroove. Condensates with minimal lateral movement were selected for side-view visualization. Scale bar: 5 µm. (MP4)

S2 Video Lateral movement of elongated condensates on microgrooves in the presence of 4% DGS-NTA. Membranes were supplemented with 4% DGS-NTA. Planar views were imaged 1 µm above the top of the membrane-clad (magenta) microgrooves using laser scanning confocal microscopy. Brighter fluorescent signals (yellow) indicate condensates located on the upper level of the membrane topographies, while areas with weaker fluorescence correspond to condensates within the grooves that are out of focus. Three-dimensional visualization of the lateral movement of elongated biomolecular condensates on microstructured lipid bilayers was reconstructed from fluorescent laser scanning microscopy images. Scale bar: 20 µm. (MP4)

S1 File Additional experimental details, materials, and methods. Supplementary information on cloning of plasmids and protein purification and FRAP experiments.
(DOCX)

## References

[1] Banani SF, Lee HO, Hyman AA, Rosen MK. Biomolecular condensates: organizers of cellular biochemistry. Nat Rev Mol Cell Biol. 2017;18(5):285–98. doi: 10.1038/nrm.2017.7 28225081 PMC7434221

[2] Li P, Banjade S, Cheng H-C, Kim S, Chen B, Guo L, et al. Phase transitions in the assembly of multivalent signalling proteins. Nature. 2012;483(7389):336–40. doi: 10.1038/nature10879 22398450 PMC3343696

[3] Elbaum-Garfinkle S, Kim Y, Szczepaniak K, Chen CCH, Eckmann CR, Myong S. The disordered P granule protein LAF-1 drives phase separation into droplets with tunable viscosity and dynamics. Proc Natl Acad Sci. 2015;112(23):7189–94. 26015579 10.1073/pnas.1504822112 PMC4466716

[4] Beutel O, Maraspini R, Pombo-García K, Martin-Lemaitre C, Honigmann A. Phase Separation of Zonula Occludens Proteins Drives Formation of Tight Junctions. Cell. 2019;179(4):923-936.e11. doi: 10.1016/j.cell.2019.10.011 31675499

[5] Gammons MV, Renko M, Johnson CM, Rutherford TJ, Bienz M. Wnt Signalosome Assembly by DEP Domain Swapping of Dishevelled. Mol Cell. 2016;64(1):92–104. doi: 10.1016/j.molcel.2016.08.026 27692984 PMC5065529

[6] Zeng M, Shang Y, Araki Y, Guo T, Huganir RL, Zhang M. Phase Transition in Postsynaptic Densities Underlies Formation of Synaptic Complexes and Synaptic Plasticity. Cell. 2016;166(5):1163-1175.e12. doi: 10.1016/j.cell.2016.07.008 27565345 PMC5564291

[7] Su X, Ditlev JA, Hui E, Xing W, Banjade S, Okrut J, et al. Phase separation of signaling molecules promotes T cell receptor signal transduction. Science. 2016;352(6285):595–9. doi: 10.1126/science.aad9964 27056844 PMC4892427

[8] Snead WT, Jalihal AP, Gerbich TM, Seim I, Hu Z, Gladfelter AS. Membrane surfaces regulate assembly of ribonucleoprotein condensates. Nat Cell Biol. 2022;24(4):461–70. doi: 10.1038/s41556-022-00882-3 35411085 PMC9035128

[9] Liao Y-C, Fernandopulle MS, Wang G, Choi H, Hao L, Drerup CM, et al. RNA Granules Hitchhike on Lysosomes for Long-Distance Transport, Using Annexin A11 as a Molecular Tether. Cell. 2019;179(1):147-164.e20. doi: 10.1016/j.cell.2019.08.050 31539493 PMC6890474

[10] Brangwynne CP, Eckmann CR, Courson DS, Rybarska A, Hoege C, Gharakhani J, et al. Germline P granules are liquid droplets that localize by controlled dissolution/condensation. Science. 2009;324(5935):1729–32. 19460965 10.1126/science.1172046

[11] Wiegand T, Liu J, Vogeley L, LuValle-Burke I, Geisler J, Fritsch AW. Proc Natl Acad Sci. 2024;121(50):e2407497121. doi: 10.1073/pnas.2407497121 PMC11648614 39630867

[12] Case LB, Ditlev JA, Rosen MK. Regulation of Transmembrane Signaling by Phase Separation. Annu Rev Biophys. 2019;48:465–94. doi: 10.1146/annurev-biophys-052118-115534 30951647 PMC6771929

[13] Agudo-Canalejo J, Schultz SW, Chino H, Migliano SM, Saito C, Koyama-Honda I, et al. Wetting regulates autophagy of phase-separated compartments and the cytosol. Nature. 2021;591(7848):142–6. doi: 10.1038/s41586-020-2992-3 33473217

[14] Zhao YG, Zhang H. Phase separation in membrane biology: The interplay between membrane-bound organelles and membraneless condensates. Dev Cell. 2020;55(1):30–44. 32726575 10.1016/j.devcel.2020.06.033

[15] Kang K, Shi Q, Wang X, Chen YG. Dishevelled phase separation promotes Wnt signalosome assembly and destruction complex disassembly. J Cell Biol. 2022;221(12):e202205069. doi: 10.1083/jcb.202205069 PMC9811998 36342472

[16] Doan VS, Alshareedah I, Singh A, Banerjee PR, Shin S. Diffusiophoresis promotes phase separation and transport of biomolecular condensates. Nat Commun. 2024;15(1):7686. doi: 10.1038/s41467-024-51840-6 39227569 PMC11372141

[17] Wu X, Ganzella M, Zhou J, Zhu S, Jahn R, Zhang M. Vesicle Tethering on the Surface of Phase-Separated Active Zone Condensates. Mol Cell. 2021;81(1):13-24.e7. doi: 10.1016/j.molcel.2020.10.029 33202250

[18] Kang CY, Chang Y, Zieske K. Lipid Membrane Topographies Are Regulators for the Spatial Distribution of Liquid Protein Condensates. Nano Lett. 2024;24(15):4330–5. doi: 10.1021/acs.nanolett.3c04169 38579181 PMC11036382

[19] Stachowiak JC, Schmid EM, Ryan CJ, Ann HS, Sasaki DY, Sherman MB, et al. Membrane bending by protein-protein crowding. Nat Cell Biol. 2012;14(9):944–9. doi: 10.1038/ncb2561 22902598

[20] Mangiarotti A, Chen N, Zhao Z, Lipowsky R, Dimova R. Wetting and complex remodeling of membranes by biomolecular condensates. Nat Commun. 2023;14(1):2809. doi: 10.1038/s41467-023-37955-2 37217523 PMC10203268

[21] Krainer G, Welsh TJ, Joseph JA, Espinosa JR, Wittmann S, de Csilléry E, et al. Reentrant liquid condensate phase of proteins is stabilized by hydrophobic and non-ionic interactions. Nat Commun. 2021;12(1):1085. doi: 10.1038/s41467-021-21181-9 33597515 PMC7889641

[22] Mangiarotti A, Siri M, Tam NW, Zhao Z, Malacrida L, Dimova R. Biomolecular condensates modulate membrane lipid packing and hydration. Nat Commun. 2023;14(1):6081. doi: 10.1038/s41467-023-41709-5 37770422 PMC10539446

[23] Kusumaatmaja H, May AI, Feeney M, McKenna JF, Mizushima N, Frigerio L. Wetting of phase-separated droplets on plant vacuole membranes leads to a competition between tonoplast budding and nanotube formation. Proc Natl Acad Sci. 2021;118(36):e2024109118. doi: 10.1073/pnas.2024109118 PMC8433588 34475202

[24] Gouveia B, Kim Y, Shaevitz JW, Petry S, Stone HA, Brangwynne CP. Capillary forces generated by biomolecular condensates. Nature. 2022;609(7926):255–64. doi: 10.1038/s41586-022-05138-6 36071192

[25] McMahon HT, Boucrot E. Membrane curvature at a glance. J Cell Sci. 2015;128(6):1065–70. doi: 10.1242/jcs.114454 25774051 PMC4359918

[26] Brangwynne CP, Mitchison TJ, Hyman AA. Active liquid-like behavior of nucleoli determines their size and shape in Xenopus laevis oocytes. Proc Natl Acad Sci. 2011;108(11):4334–9. 21368180 10.1073/pnas.1017150108 PMC3060270

[27] Xu W, Lan Z, Peng B, Wen R, Chen Y, Ma X. Directional movement of droplets in grooves: suspended or immersed?. Sci Rep. 2016;6(1):18836. doi: 10.1038/srep18836 26743167 PMC4705533

[28] Han T, Noh H, Park HS, Kim MH. Effects of wettability on droplet movement in a V-shaped groove. Scientific Reports. 2018;8(1):16013. doi: 10.1038/s41598-018-34412-0 30375434 PMC6207755

[29] Yada S, Lacis U, van der Wijngaart W, Lundell F, Amberg G, Bagheri S. Droplet Impact on Asymmetric Hydrophobic Microstructures. Langmuir. 2022;38(26):7956–64. 35737474 10.1021/acs.langmuir.2c00561 PMC9261186

[30] Starinskiy S, Starinskaya E, Miskiv N, Rodionov A, Ronshin F, Safonov A. Spreading of Impacting Water Droplet on Surface with Fixed Microstructure and Different Wetting from Superhydrophilicity to Superhydrophobicity. Water. 2023;15(4):719.

[31] Zhao JZ, Xia J, Brangwynne CP. Chromatin compaction during confined cell migration induces and reshapes nuclear condensates. Nat Commun. 2024;15(1):9964. doi: 10.1038/s41467-024-54120-5 39557835 PMC11574006

[32] Smokers IBA, Spruijt E. Quantification of biomolecular condensate volume reveals network swelling and dissolution regimes during phase transition. Biomacromolecules. 2025;26(1):363–73. 39620362 10.1021/acs.biomac.4c01201 PMC11733949

[33] Yan X, Qin Y, Chen F, Zhao G, Sett S, Hoque MJ. Laplace pressure driven single-droplet jumping on structured surfaces. ACS Nano. 2020;14(10):12796–809. doi: 10.1021/acsnano.0c05945 33052666

[34] Chu F, Yan X, Miljkovic N. How superhydrophobic grooves drive single-droplet jumping. Langmuir. 2022;38(14). 10.1021/acs.langmuir.2c00373 35348343

[35] Kim S, Kalappurakkal JM, Mayor S, Rosen MK. Phosphorylation of nephrin induces phase separated domains that move through actomyosin contraction. Molecular Biology of the Cell. 2019;30(24):2996–3012. 31599693 10.1091/mbc.E18-12-0823 PMC6857567

[36] Ghosh A, Kota D, Zhou H-X. Shear relaxation governs fusion dynamics of biomolecular condensates. Nat Commun. 2021;12(1):5995. doi: 10.1038/s41467-021-26274-z 34645832 PMC8514506

[37] Fullenkamp DE, He L, Barrett DG, Burghardt WR, Messersmith PB. Mussel-inspired histidine-based transient network metal coordination hydrogels. Macromolecules. 2013;46(3):1167–74. doi: 10.1021/ma301791n 23441102 PMC3579674

[38] Hong K, Song D, Jung Y. Behavior control of membrane-less protein liquid condensates with metal ion-induced phase separation. Nat Commun. 2020;11(1):5554. doi: 10.1038/s41467-020-19391-8 33144560 PMC7642319

[39] Shen Y, Chen A, Wang W, Shen Y, Ruggeri FS, Aime S. The liquid-to-solid transition of FUS is promoted by the condensate surface. Proc Natl Acad Sci. 2023;120(33):e2301366120. doi: 10.1073/pnas.2301366120 PMC10438845 37549257

[40] Lee S, Vörös J. An aqueous-based surface modification of poly(dimethylsiloxane) with poly(ethylene glycol) to prevent biofouling. Langmuir. 2005;21(25):11957–62. doi: 10.1021/la051932p 16316138

[41] Poudyal M, Patel K, Gadhe L, Sawner AS, Kadu P, Datta D, et al. Intermolecular interactions underlie protein/peptide phase separation irrespective of sequence and structure at crowded milieu. Nat Commun. 2023;14(1):6199. doi: 10.1038/s41467-023-41864-9 37794023 PMC10550955

[42] Testa A, Spanke HT, Jambon-Puillet E, Yasir M, Feng Y, Küffner AM, et al. Surface Passivation Method for the Super-repellence of Aqueous Macromolecular Condensates. Langmuir. 2023;39(41):14626–37. doi: 10.1021/acs.langmuir.3c01886 37797324 PMC10586374

[43] Pombo-García K, Adame-Arana O, Martin-Lemaitre C, Jülicher F, Honigmann A. Membrane prewetting by condensates promotes tight-junction belt formation. Nature. 2024;632(8025):647–55. doi: 10.1038/s41586-024-07726-0 39112699 PMC11324514

[44] Schindelin J, Arganda-Carreras I, Frise E, Kaynig V, Longair M, Pietzsch T, et al. Fiji: an open-source platform for biological-image analysis. Nat Methods. 2012;9(7):676–82. doi: 10.1038/nmeth.2019 22743772 PMC3855844

[45] Bolte S, Cordelières FP. A guided tour into subcellular colocalization analysis in light microscopy. J Microsc. 2006;224(Pt 3):213–32. doi: 10.1111/j.1365-2818.2006.01706.x 17210054

[46] Schmid B, Schindelin J, Cardona A, Longair M, Heisenberg M. A high-level 3D visualization API for Java and ImageJ. 2010. 10.1186/1471-2105-11-274 PMC2896381 20492697

