# Supplementary figures and images for: Topography-driven movement of biomolecular condensates
Source: PLoS One. 2026 Apr 15;21(4):e0345319. doi: 10.1371/journal.pone.0345319 (PMC13082584; doi:10.1371/journal.pone.0345319)

A

top

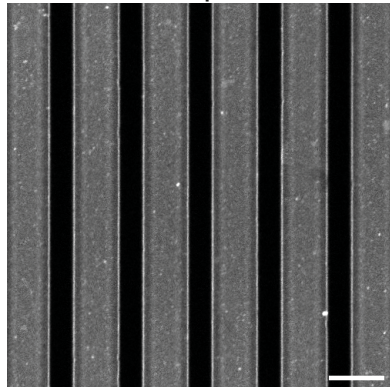

mid

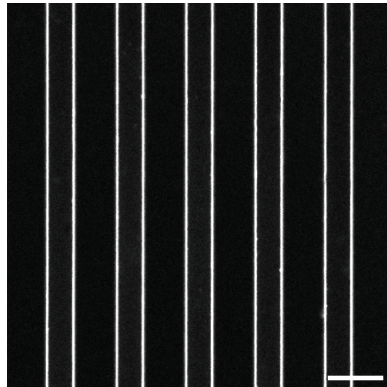

bottom

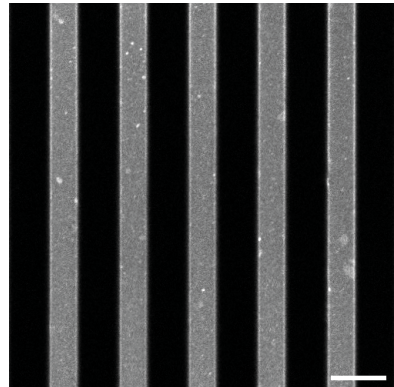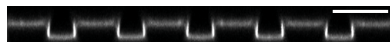

B

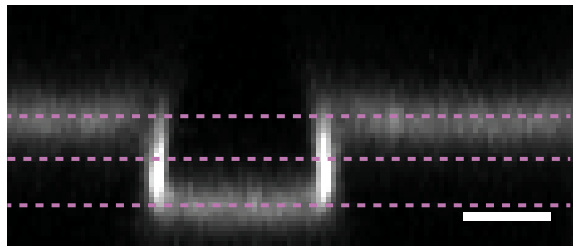

top

mid

bottom

C

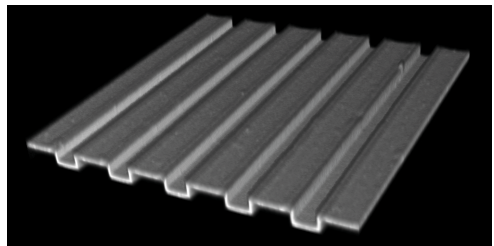

Supplement: S1 Fig — (A) Fluorescent laser scanning confocal microscopy images of the lipid bilayer, labelled with 0.05 mol% Fast DiI. Images show planar views (top, mid and bottom of the microstructure) and a side view. Scale bars: 20 µm. (B) Side view of a membrane-clad microstructure, with annotations for the levels defined as “top”, “mid” and “bottom”. Scale bar: 5 µm. (C) 3D reconstruction of the lipid bilayer topography, derived from z-stack images obtained via fluorescent laser scanning confocal microscopy. (PDF) [file pone.0345319.s001.pdf]

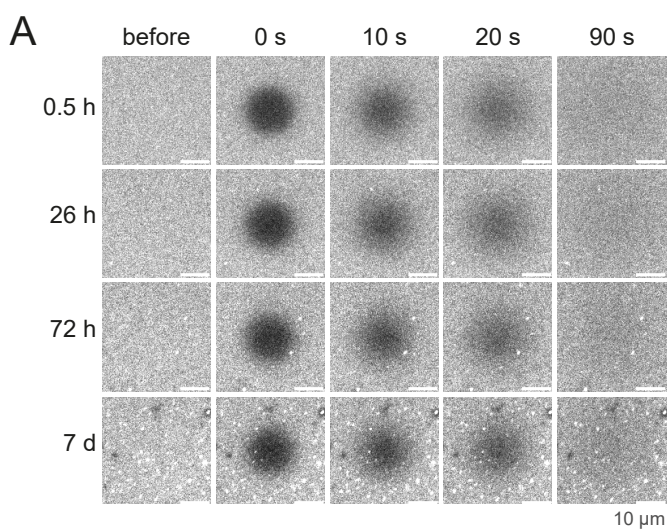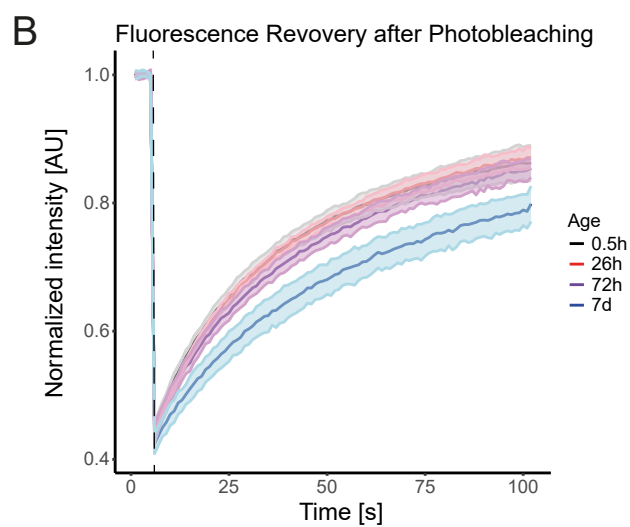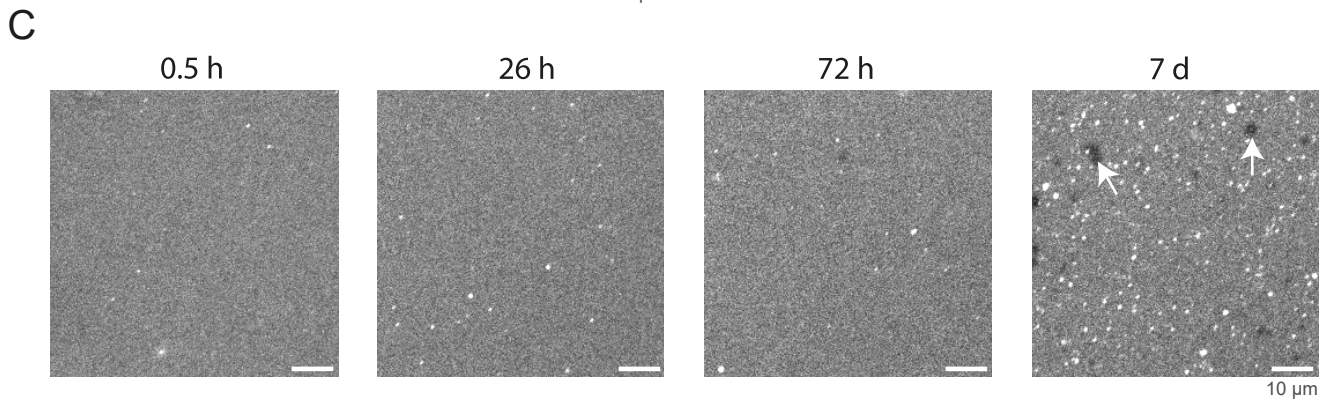

Supplement: S2 Fig — (A) FRAP experiments were performed at various time points (30 min, 26 h, 72 h, and 7 d after membrane formation) on membranes composed of DOPC and 0.05 mol% Fast DiI. (B) Fitted FRAP curves and quantification of half-time recovery reveal stable membranes over 26h (t1/2–30min: 21.6 ± 1.6 s; t1/2-26h: 22.7 ± 1.0 s), with slightly reduced fluidity after 3 days (t1/2-72h: 25.8 ± 0.6 s) and more pronounced reduction of fluidity after seven days (t1/2-7d: 31.2 ± 2.1 s). Dashed line: time point of bleaching of the membrane. Half-time recovery and line plot show the mean intensity ± standard deviation. n = 9 from three independent samples. (C) Membrane homogeneity is maintained within 72 h, but impaired after 7 d, with white spots and darker areas indicating compromised membrane integrity. (PDF) [file pone.0345319.s002.pdf]

**A**

0% DGS-NTA

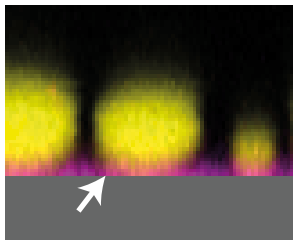

0.25% DGS-NTA

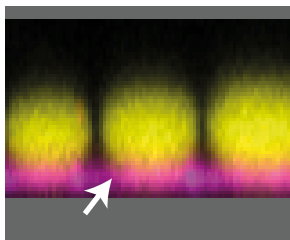

0.5% DGS-NTA

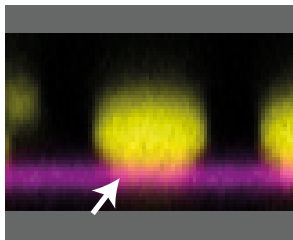

2% DGS-NTA

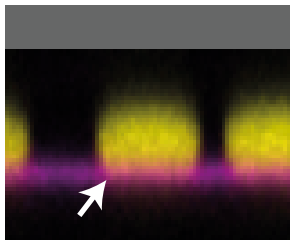**B**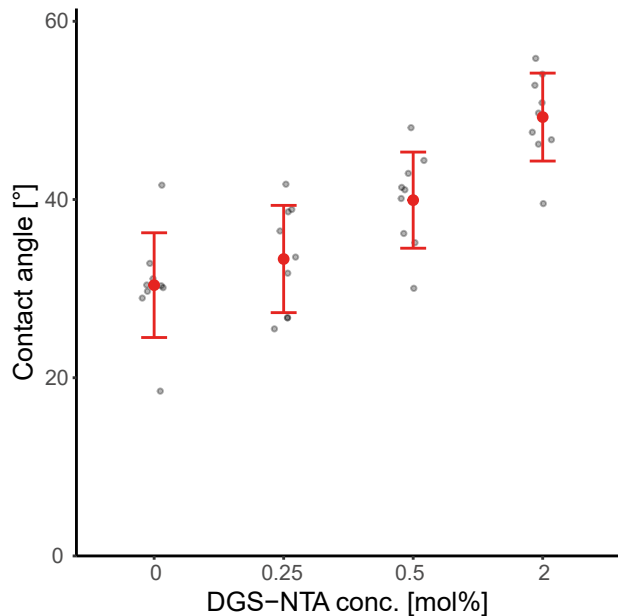

Supplement: S3 Fig — (A) Confocal fluorescence microscopy images of biomolecular condensates (yellow), chosen to exhibit an average xy-diameter of 7.7 ± 0.5 µm, formed on DOPC lipid membranes containing 0.05 mol% Fast-DiI (magenta) and supplemented with 0 mol%, 0.25 mol%, 0.5 mol%, or 2 mol% DGS-NTA. Condensates wet the lipid membrane with higher DGS-NTA concentrations with an increasing contact angle (white arrow). Independent samples: n = 3. Protein concentrations: 40 µM PRM4, 40 µM SH34-6xHis. Scale bar: 10 µm. (B) Quantification of contact angle using ImageJ’s contact angle plugin. Individual measurements per condensate were depicted (black data points) alongside the mean value ± standard deviation (red). (PDF) [file pone.0345319.s003.pdf]

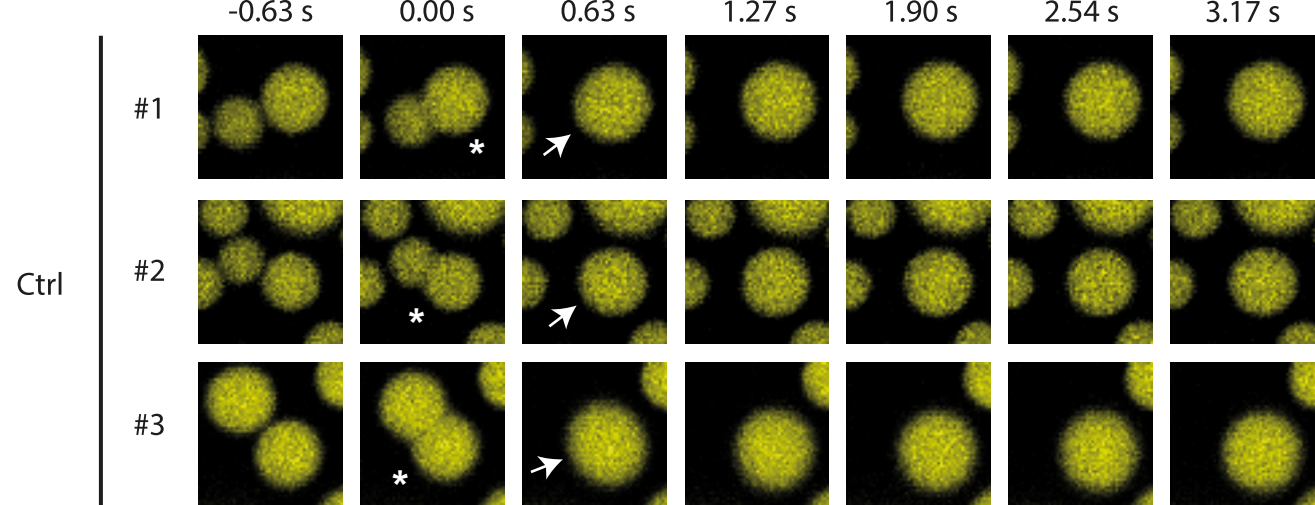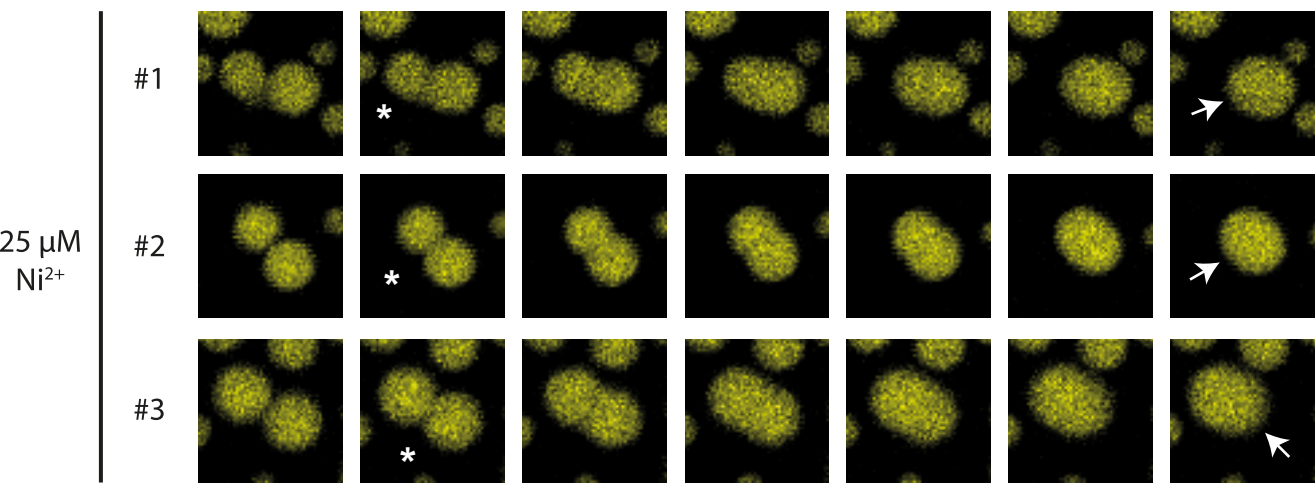

Supplement: S4 Fig — Fusion assay with and without 25 µM Ni2+ was performed 70 ± 5 min after condensate formation was initiated by supplementing samples with 40 µM PRM4 and 40 µM SH34-6xHis. Image sequence shows representative fusion events for n = 3 independent replicates. The time point t = 0 refers to the onset of fusion of two similar sized condensates. Asterisks indicate the onset, and arrows the end of fusion events. Lipid bilayer composition: DOPC, 0.05 mol% Fast-DiI. Scale-bar: 10 µm. (PDF) [file pone.0345319.s004.pdf]

Size of biomolecular condensates over time

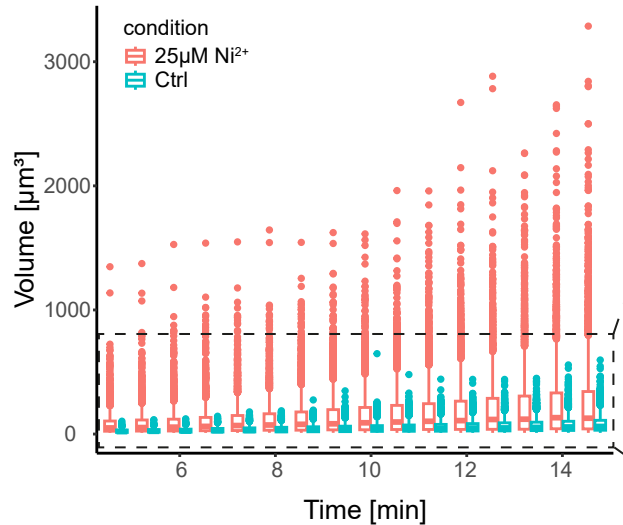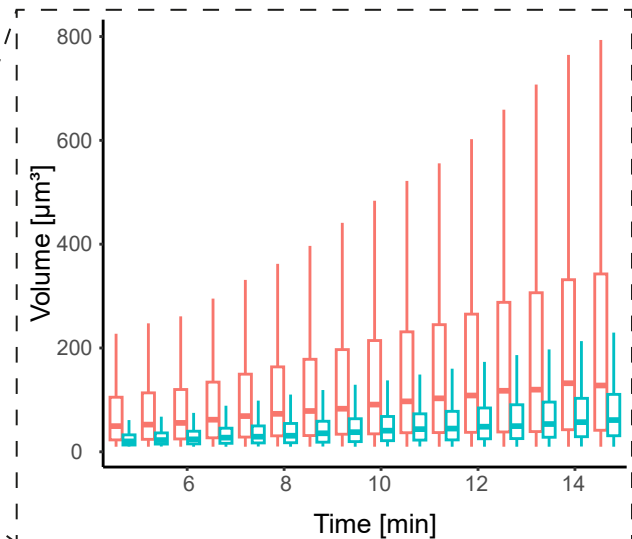

Supplement: S5 Fig — Boxplots show size analysis of biomolecular condensates larger than 10 µm3 in the first 5 to 15 min after their formation in the presence and absence of 25 µM Ni2+. The right panel shows boxplots after exclusion of outliers (defined as outside median±1.58*interquartile range (IQR)n). (PDF) [file pone.0345319.s005.pdf]
