# Supplementary material for: Topography-driven movement of biomolecular condensates
Source: PLoS One. 2026 Apr 15;21(4):e0345319. doi: 10.1371/journal.pone.0345319 (PMC13082584; doi:10.1371/journal.pone.0345319)
Supplement: S1 File — Supplementary information on cloning of plasmids and protein purification and FRAP experiments. (DOCX) [file pone.0345319.s008.docx]

**ADDITIONAL EXPERIMENTAL DETAILS, MATERIALS, AND METHODS**

**Cloning of plasmids**

The plasmid encoding for SH3_4_-6xHis was generated by Gibson assembly from pGEX SH3(2)-4R (Addgene #112090) (1). A high-fidelity PCR was performed using the following primers: 5’-tgtcatcatcatcatcatcactgaaggatccgcggccgcatcgt-3’ (forward) and 5’-cctcagtgatgatgatgatgatgacacaaaggactgtcaccttcttcagt-3’ (reverse). The amplified product was run on a 0.66% agarose gel. DNA was isolated from the band of the desired size. The linear DNA was annealed using the Gibson assembly cloning kit (New England Biolabs) and bacteria (DH5alpha, New England Biolabs) were transformed with this plasmid. Colony PCR and Sanger sequencing confirmed the correct sequence. The plasmid encoding for PRM_4_ (pMAL-Abl-PRM 4R, Addgene #112087) was previously mutated to introduce a cysteine tag for labeling (2).

**Protein purification**

Purification protocols were adapted from previously published protocols (1). Proteins were expressed in BL21(DE3) pLysS Competent Cells (Agilent Technologies, #200132). Transformed bacteria were grown at 37°C in Terrific Broth medium containing 0.1 mg/ml ampicillin. Protein expression was induced by adding 1 mM IPTG at an OD between 0.6 and 0.8. Bacterial suspensions were then incubated overnight at 18°C.

**Purification of SH3_4_-6xHis**: Bacteria were pelleted, resuspended in GST-Trap buffer A (pH=8.0, 50 mM Tris, 75 mM NaCl, 1 mM DTT) and lysed by sonication on ice. Lysates were cleared by centrifugation at 15,000g for 30 min at 4°C.

Cleared lysates of SH3_4_-6xHis producing bacteria were incubated on equilibrated Pierce™ Glutathion-Agarose (Thermo Scientific) at 4°C for 3 hours to overnight. Protein was eluted using GST-Trap buffer B (pH=8.0, 50 mM Tris, 75 mM NaCl, 1 mM DTT, 25 mM reduced glutathione) and the GST-tag was cleaved with TEV protease at room temperature for 2-3 hours. The cleaved products were further purified on a HiResQ anion exchange column (Cytiva). The column was equilibrated with HiResQ wash buffer (pH=8.0, 50 mM Tris, 1 mM DTT, 100Mm NaCl), loaded with the protein solution, and washed with HiResQ wash buffer. Protein was eluted in a gradient elution over 30 column volumes in HiResQ buffer B (pH=8.0, 50 mM Tris, 1 mM DTT, 1 M NaCl).

**Purification of PRM_4_**: Bacteria were pelleted, resuspended in HiTrap buffer A (pH=7.5, 50 mM phosphate buffer, 100 mM NaCl, 20 mM imidazole) and lysed by sonication on ice. Lysates were cleared by centrifugation at 15,000g for 30 min at 4°C. Lysates were applied to a HiTrap Chelating HP column (Cytiva), washed, and eluted in HiTrap B Buffer (pH=7.5, 50 mM phosphate buffer, 100 mM NaCl, 500 mM imidazole). Proteins in the eluate were cleaved with TEV protease overnight on ice. Cleaved products were separated on a HiResS cation exchange column (Cytiva). The column was equilibrated with HiResS wash buffer (pH=7.5, 50 mM phosphate buffer, 1 mM DTT, 100 mM NaCl), loaded with protein solution, washed with HiResS wash buffer and gradient eluted over 10 column volumes in elution buffer (pH=7.5, 50 mM phosphate buffer, 1 mM DTT, 1 M NaCl). For PRM_4_ labelling, PRM_4_ containing a cysteine tag for labelling was concentrated to 100 µM and incubated with 2 mM Alexa Fluor™ C_5_ reactive dye for 2 hours at room temperature. The residual dye was removed using a PD-10 Desalting Column (Cytiva).

The buffer was exchanged by dialysis in KMEI buffer (pH=7.5, 150 mM KCl, 1 mM MgCl_2_, 1 mM EGTA, 10 mM imidazole) at 4°C overnight. Protein solutions were concentrated to approximately 200 µM using a Vivaspin® (Sartorius). Aliquots were snap frozen in liquid nitrogen and thawed only once.

**FRAP experiments to verify fluidity of supported lipid bilayers**

FRAP assays were performed on supported lipid bilayers on top of plasma-activated PDMS surfaces. The lipid bilayers were composed of DOPC (Avanti Polar Lipids) and 0.05 mol% Fast DiI. Photobleaching of 15 µm diameter circles was performed using a 561 nm laser. The fluorescence recovery of the bleached areas was monitored through time-lapse imaging (one frame per second) (Supplementary figure S2A, B). The fluorescence intensity of the circular area before bleaching was normalized to one. The half-time recovery was calculated by fitting the fluorescence recovery data to an inverse negative exponential decay curve using a non-linear regression analysis. Three regions were analyzed for three independent samples.

$median\pm1.58*\frac{interquartile range (IQR)}{\sqrt{n}}$

Sources:

1. Li P, Banjade S, Cheng HC, Kim S, Chen B, Guo L, u. a. Phase transitions in the assembly of multivalent signalling proteins. Nature. März 2012;483(7389):336–40.

2. Kang CY, Chang Y, Zieske K. Lipid membrane topographies are regulators for the spatial distribution of liquid protein condensates. Nano Lett. 17. April 2024;24(15):4330–5.
